# Exploring the lived experiences of the *suicide attempt* survivors: a phenomenological approach

**DOI:** 10.1080/17482631.2020.1745478

**Published:** 2020-03-29

**Authors:** Farshid Shamsaei, Safura Yaghmaei, Mohammad Haghighi

**Affiliations:** a Behavioral Disorders and Substance Abuse Research Center, Hamadan University of Medical Sciences, Hamadan, Iran; b Department of Nursing, School of Nursing & Midwifery, Hamadan University of Medical Sciences, Hamadan, Iran; c Department of Nursing, School of Nursing & Midwifery, Semnan University of Medical Sciences, Semnan, Iran

**Keywords:** Lived experiences, suicide attempt, phenomenology, mental pain, belonging

## Abstract

**Purpose**: Suicide is a complex phenomenon that needs to be studied with a variety of approaches. The purpose of this study is to explore the lived experience of attempted suicide with the phenomenology approach.

**Method**: An interpretive phenomenological approach was used to analyse semi-structured, in-depth interviews with 16 participants (ages 19–57) who were recruited by means of purposive sampling from October to November 2017 at the Farshchian Psychiatric Hospital in Hamadan/Iran. Data analysis was conducted according to van Manen’s phenomenological method.

**Results**: Identified three themes and eight subthemes: Mental pain (subthemes: living through grief, internal conflict, the world is better without me), Social challenges (lack of social connection, financial problems, social support services) and Need for love and belonging (feeling *understood*, need of empathy).

**Conclusions**: Outcomes and key implications of the study related primarily to improving the treatment experiences of suicide attempt survivors and other at-risk population, and also advancing suicide prevention efforts and to provide support for suicide attempt survivors.

## Introduction

Suicide is a global public health problem. Worldwide, suicide is the fifteenth leading cause of death, accounting for 1.4% of all deaths (World Health Organization [WHO], 2014). It is estimated that suicides claim approximately 1 million lives worldwide every year, and as many as 60% occur in Asia (Beautrais, 2006; Chen et al., 2012). The annual global age-standardized death rate for 2012 is estimated to be 11.4 per 100,000, and the World Health Organization (WHO) projects this rate to remain steady through 2030 (WHO, 2014). In addition to suicide deaths, suicidal thoughts and nonfatal suicide attempts also warrant attention. Globally, lifetime prevalence rates are approximately 9.2% for suicidal ideation and 2.7% for suicide attempt (Nock et al., 2010).

Suicide ideation and attempts are strongly predictive of suicide deaths; can result in negative consequences such as injury, hospitalization, and loss of liberty; and exert a financial burden of billions of dollars on society (Nock et al., 2013; WHO, 2014). Taken together, suicide and suicidal behaviour comprise the nineteenth leading cause of global disease burden (i.e., years lost to disability, ill-health, and early death), and the sixth and ninth leading cause of global disease burden among men and women 15 to 44 years of age, respectively (World Health Organ [WHO], 2008). By any measure, there is urgency to better understand and prevent suicide and suicidal behaviour (Klonsky et al., 2016; WHO, 2008).

Although the rate of suicide is low in most Islamic countries, evidence shows that it is increasing (Pritchard & Amanullah, 2007; WHO, 2014). In recent years, suicide has become a serious mental health problem in Iran. In Iran, the suicide rates per 100 000 people are 5·3 in both sexes, 3·6 in females, and 7·0 in males (Nazarzadeh et al., 2013; Pritchard & Amanullah, 2007).

Suicide is a complex, multidimensional phenomena that has been studied from philosophical, sociological, and clinical perspective. Suicidal behaviour can be conceptualized as a continuum ranging from suicidal ideation to suicide attempts and completed suicide. Attempted suicide is defined as a potentially self-injurious action with a nonfatal outcome for which there is evidence, either explicit or implicit that the individual intended to kill himself or herself. The action may or may not result in injuries (Ramdurg et al., 2011).

Lakeman and Fitzgerald systematically reviewed the existing literature to determine how people lived with their suicide ideation, especially in terms of “recovering a desire to live” (Lakeman & Fitzgerald, 2014). They found that people struggled with suicide ideation, and often described disconnection from others and intense suffering. A more recent review highlighted the balance people had to make between the intense suffering of continuing suicidal ideation and hope for the future after surviving a suicide attempt (Berglund et al., 2016).

Similarly, in another study, reported that only 10 of the 50 participants in their study reported feeling happiness or relief immediately after surviving their suicide attempt. The predominant feelings reported were negative including feeling sad, depressed, disappointed, empty, angry, embarrassed or ashamed. While these negative emotions had changed for many participants by the time of the participation in the study, to reports of feelings of gladness, gratefulness, and hope, over 30% of participants continued to feel negatively towards their survival (Maple et al., 2019).

Attempts to explain, predict and prevention suicide require an understanding of what suicidal thoughts and feelings mean to those who live it (Pompili, 2010). To understand any human experience we must first ask the phenomenological question, “What is this or that kind of experience like?” (Van Manen, 1997). Although the early pioneers of suicidology, like Edwin S. Shneidman and Erwin Stengel, asked “What is it like to be suicidal?” the trend in recent decades has been for the phenomenology of suicidality to almost disappear from the research agenda of the discipline (Pompili, 2010).

Unlike epidemiological studies of suicide risk, qualitative case studies can help to explain the social and cultural context of suicidality among Iranian people and how those affected by suicidality make meaning of these experiences. Understanding the subjective, lived experiences of those affected by suicidality has important potential to inform the innovation of culturally appropriate health services, shape public policy, and promote meaningful social change (Shaw et al., 2019). This paper reports on an interpretive phenomenological qualitative study that explored the lived experiences of the suicide attempt survivors.

## Methods

### Study design

This study was a qualitative research with interpretive hermeneutic phenomenological approach that was conducted from February to September 2017. interpretive hermeneutic phenomenological is a qualitative methodology used to examine and understand the subjective, lived experience of individuals using an idiographic approach, in which detailed, individual cases are used to develop more general claims (Paley, 2018). As Smith and Osborn point out, interpretive hermeneutic phenomenological is “especially valuable when examining topics which are complex, ambiguous and emotionally laden”, such as suicide (Smith & Osborn, 2008).

### Setting

The research was carried out in Hamedan city, with in the west region of Iran. The city has available the services of about 12 psychiatrists and 200 psychiatric beds and four psychiatric wards in Farshchian psychiatric hospital, and one public mental health services for children. This hospital is the oldest teaching mental hospital in Iran and is located in the centre of the city. Also, this hospital has the highest referral level and all their facilities are integrated with mental health outpatient facilities. People can choose freely where they want to be treated. Two researchers with experience in qualitative studies conducted the interviews in a private interview room at the psychiatric wards in Farshchian Psychiatric Hospital.

### Participants

The participants in the study were recruited by purposive sampling from the psychiatric wards at a Farshchian psychiatric hospital, and also informants who will better be able to assist with answering the research questions and are readily available to participate in the study. Inclusion criteria included: adult 18 ≥ years of age, Persian speaking, male and female, all ethnic and racial groups, attempted suicide one or more times, and admitted to the Farshchian psychiatric hospital for their attempt. The exclusion criteria included participants deemed emotionally unstable either during the inpatient stay or after discharge as determined by their mental health providers. In addition, patients diagnosed with a psychotic disorder or other cognitive disorder that would make interviewing difficult were excluded.

Sixteen adult participants were recruited into this study, as this was the number at which “saturation” of the data was reached. After the 14th interview, there were no new themes generated from the interviews. Therefore, it was deemed that the data collection had reached a saturation point. We continued data collection for two more interviews to ensure and confirm that there are no new themes emerging. Sample sizes in phenomenological studies have varied from 5 to 30 (Smith & Osborn, 2008).

### Data collection

Data were collected through semi-structured individual face to face interview. The interviews ranged from 40 to 60 minutes in length. Each participant was interviewed once. The interview began with an opening question, “Tell me about your experience of suicide attempt?” Next came a series of potential prompts followed by prompts: “Tell me more about?” “What was that like?” And, “Can you give me an example.

The interviewer listened and observed the participants closely, noted their body language and tone of voice. The interviews were recorded and then transcribed verbatim by a research transcriptionist educated in research ethics and in hermeneutic transcription.

### Data analysis

There are varying processes by which interpretive phenomenologists analyse data depending on the specific type of phenomenology being used. For this study purposes, we have chosen the analytical techniques described by Van Manen (1997). These six steps are:

1. Turning to a phenomenon of interest: Van Manen expresses that the first step in studying a phenomenon is the researcher’s interest in nature of that research. The researcher’s attention was drawn to the phenomenon of the lived experiences of the suicide attempt survivors.
2. Investigating experiences as we live it: Patients who referred to Farshchian Hospital in Hamadan city, Iran in 2018, underwent a purposive selection of participants with maximum variation sampling.
3. Reflecting on the essential themes which characterize the phenomenon: Thinking in inherent themes was descriptor of the considered phenomenon. Researcher determined the main themes for the phenomenon of the suicide attempt survivors. Through thematic analysis.
4. Describing the phenomenon-the art of writing and rewriting: Van Mannen’s approach is the art of writing and rewriting. In this stage, the purpose was achieving a strong description of the phenomenon of patients’ experiences with suicide attempt.
5. Maintaining a strong and orientated relation to the phenomenon: This stage is for maintenance of a strong and directional communication with the considered phenomenon. In this stage, in order to avoid getting away from the purpose of study, the researcher considered the main research question to maintain the strong and directional communication with the phenomenon.
6. Balancing the research context by considering the parts and the whole: Van Mannen is making a balance in the field of linking whole and detail. He worked on this stage by continues review of whole and detail through holistic and selective approaches and according to the main research question.

The goal of the analysis was to identify the meaning from the input, experience and understanding of all researchers analysing the data (example of the hermeneutic circle) in addition to the interviews (Van Manen, 1997).

### Evaluation and trustworthiness

Data were validated through member check and participants’ review of transcriptions. Reliability was achieved through constant analysis and devoting enough time to data collection, as well as more and longer involvement during the research. Also, consulting with colleagues was done to obtain deeper data and general understanding. Data were confirmed by the review of a foreign supervisor; also, the research group confirmed coding and categorizing processes. To achieve the criteria of transferability, it was tried to explain the process of research precisely so that other researchers can use this method. (Tracy, 2010).

### Ethics

The study was approved by the Ethics Committee of Hamadan University of Medical sciences (IR.UMSHA.REC.1396.292). An informed consent document provided to each participant explained the purpose of the research, detailed potential risks, and provided a confidentiality statement of how participant information would be securely handled. In addition, the informed consent document acknowledged that there was likelihood that the participant could feel emotional discomfort during the interview and noted that the researcher could provide a list of available suicide prevention services; or, if the participant needed to speak with a professional immediately.

## Results

Participant Background Information:

The participant sample consisted of 16 adults. The participants ranged in age from 19 to 57 years old. Four out of the 16 participants were female. Four out of the participant attempted suicide more than once (Table I).

**Table I.** Demographic characteristics of the participants

NO	Age	Sex	Marital status	Education	Occupation	No of suicide attempt	Attempt method
1	23	Male	Single	High school	Student	1	Drug ingestion
2	19	Male	Married	University	Student	1	Cutting/stabbing
3	31	Male	Divorced	Primary school	Transport worker	2	Hanging
4	29	Female	Bereaved	Middle school	Unemployed	1	Drug ingestion
5	22	Male	Separated	Middle school	Unskilled worker	1	Ingestion of other toxic substance
6	26	Male	Single	High school	Unskilled worker	3	Drug ingestion
7	21	Male	Single	High school	Student	1	Traffic accident
8	51	Female	Married	Primary school	Housewife	1	Jumping
9	45	Male	Married	Middle school	Farmer	2	Ingestion of other toxic substance
10	38	Male	Married	High school		1	Jumping
11	57	Female	Separated	Middle school	Housewife	1	Drug ingestion
12	43	Male	Married	Primary school	Unemployed	1	Drug ingestion
13	27	Male	Married	High school	Unskilled worker	3	Cutting/stabbing
15	31	Male	Married	High school	Unemployed	1	Cutting/stabbing
16	20	Female	Single	Primary school	Housewife	1	Ingestion of other toxic substance

The main themes were illuminated from the text of the 16 participants in the research study. That is, the themes in this study were the expressive parts of the participants’ experience that portrayed the understanding as a whole.

The careful evaluation of the themes that emerged from the participant stories helped to understand the Suicide attempters’ experiences regarding the phenomenon.

The following three themes emerged and discussed in the details: 1. Mental pain 2. Social challenges 3. Need for love and belonging (Table II).

**Table II.** Themes identified and subthemes

Themes	Subthemes
Mental pain	Living through grief, Internal conflict, the world is better without me
Social challenges	Lack of social connection, financial problems and social support services
Need for love and belonging	Feeling *understood*, need of empathy

### Mental pain

Mental pain is one of the main facilitators of suicide. Participants’ experiences have shown that mental pain are characterized by high levels of hopelessness, Grief and mental suffering. This theme is described with three subthemes: Living through grief, internal conflict and the world is better without me.

#### Living through grief: many contributors expressed sadness, depression and emptiness

One of the participants said: “I Hate My Life. I find too much time alone is unhelpful, even though sometimes I just don’t want to leave the house or see anyone … I have no desire to do anything. I don’t enjoy my life at all …. I Have No Motivation To Do Anything”. (23-year- girl)

A 31-year-man says: “My attempt had nothing to do with how ‘good’ or ‘bad’ my life is. It came from being tired. Tired of being me, tired of pretending, tired of being depressed. The emotional pain we feel becomes physical and it feels like there is no light at the end of our tunnel.”

#### Internal conflict: suicide attempters are sometimes exposed to internal contradictions

One of the participants said: “I don’t want to get this thing out again and again and talk about it. It is embarrassing that I attempted suicide … . I feel not good. It is because I have difficulty in expressing my feelings in interacting with others. When I see myself now doing it I am ashamed. I can express my feeling only poorly. Seeing myself it is much worse”. (27-year- male)

A participant says: “People think it all about wants to die. No, it isn’t. I’m quite scared of death, if I’m totally honest, but I faced that fear because it [felt] easier than living a lifetime full of pain and exhaustion. It seems like the best and only way out at the time.” (38-year- male)

A 29-year-man says “I believed my existence was doing more harm to those around me than good. I believed the pain of dealing with my death would be temporary, but if I stayed I would cause more harm to those I loved. It was not a cry for attention. I just saw no other way”.

#### The world is better without me: some participants said the world would be better without them

One of the participants says: “The world would be better off without me. I cause everyone more stress and pain and suffering. And worst off, I cannot express my pain and suffering because it will just cause everyone else more stress. I cannot take this anymore. This kind of pressure, stress and anxiety is eating me alive”. (51-year- female)

Another participant says: “I have [attempted suicide] numerous times and am not ashamed to admit it anymore because yes, sometimes I feel like I don’t want to be here or that people are better off without me.” (27-year- male)

A 21-year-man says: “I truly believed I was doing what was best for my family. When people say that suicide is selfish it bothers me. I can honestly say I wasn’t thinking at all about myself”.

### Social challenges

Suicidal behaviour to be in some way related to social factors of cohesion and integration. Suicide attempters suffer from several problems, some of which are related to society and social factors. This theme is described with three subthemes: Lack of social connection, financial problems and social support services.

A. Lack of social connection: Social connections are potential protective factors for suicide. Lower levels of social connections were associated with an increased risk of suicide attempt. The majority of participants said that a general lack of support was a causal factor in their attempts.

“A 43-year male believes suicidality stems from social problems. When asked to share her experiences, she responds: “since my relationship with my friends has diminished, I am less out of the house and more alone. I am not motivated to do anything. I think I’m forgetter because no one cares for me in the community. The situation is such that I have less connection with my brother and sister so I think this makes me separate from people”.

A student participant says: “If I had had people to check in with on a regular basis that would have helped. Um, if I had had just someone to talk to. I didn’t really because I was new in the dorm, I didn’t have many friends yet.”

B. Financial problems: One of the participants described how stress related to finances contributed to their attempts: My problems are high and the financial crisis has made everything worse. I lost my job. I have no money, I am ashamed of my family, and I can’t meet their needs. I tried to find a way to get income, but no use and the financial problems of the day increased and I said I would die and get rid of it. (38-year-male)

#### Social support services: social support emerged as a suicide protective factor in the current study

I needed help because I had a lot of tensions and needed support … something is really wrong with our mental health system. Like, there’s lack of resources, now here to go … (22-year-old woman)

“One participant stated, “I sought professional help myself—but that was only very recently. I wish there had been some sort of counselling or service that offered me assistance … But it was not easy to access”.

### Need for love and belonging

The need to belong, also often referred to as belongingness, refers to a human emotional need to affiliate with and be accepted by members of a group. This may include the need to belong to a peer group at school, to be accepted by co-workers, to be part of an athletic team and to be part of a church group. It involves more than simply being acquainted with other people. It is instead centred on gaining acceptance, attention, and support from members of the group as well as providing the same attention to other members. This theme is described with two subthemes: feeling understood and need of empathy.

#### *Feeling* understood*: some participants have spoken about their need for understanding*

A 21 year old boy student says: *“Attempting wish others understood I am not a danger to them. After my attempt, my friends kept me at arm’s length rather than drawing close to me because they were afraid I would hurt them too. It left me feeling more isolated and rejected than ever. I also wish people understood the power of what seems like a simple little thing. A hug. A text. A phone call, even if I can’t bear to answer and you get my voice mail. Tiny little things that are actually huge things because they say, ‘I want you here, I want to help you fight*.”

#### Need of empathy: empathy was another participant experience

A 29-year-man says: “How could you ever give up on life?” They don’t understand the fact that the will of a suicide is more than just a simple desire. Even though you try not to think about it, even though you don’t want to do it, there is this strong and hopeless feeling of just … doing it.”

“Do you understand how much it hurts to be criticized for having this in our past? Do you know how much it hurts to be called ‘selfish,’ ‘stupid’ and ‘crazy?’ If you have never had suicidal ideation, please do not place judgement on those of us who have, because of course it doesn’t make sense to you … it doesn’t make sense to most of us either. Unfortunately it still happens, and we deserve help, not hate.” (20-year- female)

## Discussion

The purpose of this study was to understand the lived experiences of the suicide attempt survivors. One of the main themes of this study was mental pain. Suicide is multifaceted and rarely the result of any single cause. However, suicide risk is most commonly associated with mental illness. Mental disorders play an overwhelming role in the increased risk of suicide—with estimates suggesting up to 90% of individuals who take their own life suffer from some type of psychiatric disorder (Phillips, 2010).

Several studies conducted with suicide attempters’ patients have shown significant associations between mental pain and suicidality. Berlim et al. (2003) reported a significant association between psychache and suicidality with a correlation identified as the highest in magnitude. These results were confirmed by Mee et al. (2011) using a sample of 73 outpatients with major depression compared with 96 non-psychiatric controls. Xie et al. (2014) have provided a further evidence supporting the relevance of psychological pain in the risk of suicide by demonstrating that outpatients with major depressive episodes and high levels of suicidal ideation showed anticipatory anhedonia and stronger pain avoidance matched to those with low levels of suicidal ideation and healthy controls. Caceda et al. (2014) evaluating a sample of 62 depressed patients compared with a sample of 20 healthy controls, have highlighted that psychological pain predicted the presence of suicidal ideation in the overall sample by differentiating, using logistic regression analysis, between the suicide attempt and suicidal ideation groups.

The relationship between suicidal risk and mental pain was further evaluated in a study by Gould et al. aimed at investigating the effectiveness of a telephone crisis services. Gould et al. have found a significant reduction in suicide risk factors (i.e., plans, actions, and prior attempts) with diminishing levels of psychological pain from the beginning to the end of the call (Gould et al., 2007).

Other studies investigated the mediational role of mental pain. Nahaliel et al. (2014) have shown that self-destruction causes both a direct effect on suicidal tendency, and an indirect effect mediated by the presence of mental pain. Subsequently, Campos and Holden (2015) have revealed that psychache and interpersonal needs mediate the relationship between depression and suicide risk. Mental pain seems to be a leading cause of suicide only when it is experienced as unbearable according to the cubic model of Shneidman (Verrocchio et al., 2016).

Recently, studies highlighted the clinical relevance of tolerance as a component of mental pain to explain its association with suicidality. In this regard, Levinger et al. (2015) by using a multivariate analysis of covariance (i.e., MANCOVA), have demonstrated that suicidal respondents reported higher levels of mental pain, as well as a lower tolerance for such pain compared to non-suicidal group. In a Phenomenological Study, the following salient experiences emerged during the data analysis: guilt, self-blame, blaming others or God, anger, loss or restriction of “self”, depression, suboptimal behavioural coping patterns, changes in relationship dynamics, and suicidality (Hoffmann et al., 2010).

The results indicate that levels of mental pain are associated with an increased risk of suicide. In other terms, high levels of mental pain may represent a condition of vulnerability to suicidal ideation, suicidal attempts, and suicide acts. By supporting the evidence that psychological pain plays a central role in predicting suicide, the results of this qualitative study could further underline the clinical relevance of psychache in prevention (i.e., early detection) as well as in treatment of suicide. In this regard, mental pain may represent, from a clinical point of view, an important therapeutic target, when considering that diminishing levels of psychache potentially means to decrease the risk of suicidal acts. Therefore, by identifying psychological pain in order to evaluate the suicidal risk, psychache can be targeted during clinical interventions and considered amenable to treatment.

The present study showed that social challenges were another theme. The sociocultural factors that affect suicide rates operate at many different levels. The degree to which someone’s surroundings bring about a positive or negative influence depends on individual factors. These include demographic characteristics, life stressors, coping skills, and economic status linked to suicide. They also include whether an individual’s family, community and country are supportive or stressful (Milner et al., 2015).

The results of this study are broadly consistent with previous research on social connections and Social support and suicide and attempted suicide (Kleiman & Liu, 2013). For example, in a sample of young adults attending Miami-Dade public school system, Joiner et al. (2009) documented the contribution of low family social support and the feeling “that one does not matter” to suicidal ideation. The authors interpreted these findings in terms of the Interpersonal-Psychological Theory of Suicide, which argues that two interpersonal constructs, perceived burdensomeness and thwarted belongingness “instill the desire for death” as a necessary condition for suicidal behaviour. Although suicide ideation is not attempted suicide or suicide itself, it is likely to be relatively prevalent in young people; thus, this study lends support to the idea that a greater number of social connections are protective factors for suicide.

Our findings suggest one potential resiliency factor that warrants consideration is social support. Social support is anything that leads someone to “believe that he is cared for and loved, esteemed, and a member of a network of mutual obligations. Social support is a highly modifiable factor that can be used to improve existing suicide prevention programs worldwide.

Another major theme from the experiences of the participants was the Need for love and belonging.

The need to belong, also often referred to as belongingness, refers to a human emotional need to affiliate with and be accepted by members of a group. This may include the need to belong to a peer group at school, to be accepted by co-workers, to be part of an athletic team, and to be part of a church group (Le Penne, 2017).

The need to belong involves more than simply being acquainted with other people. It is instead centred on gaining acceptance, attention, and support from members of the group as well as providing the same attention to other members. This need plays a role in a number of social phenomena such as self-presentation and social comparison. This need to belong to a group can also lead to changes in behaviours, beliefs, and attitudes as people strive to conform to the standards and norms of the group (Bailey & McLaren, 2005).

Individuals with a lower sense of belonging are more likely to report current or past suicidal thoughts or attempts than individuals with greater sense of belonging. Sense of belonging is associated with a history of suicide attempts among individuals receiving treatment for opiate dependence (Conner et al., 2007). Coupled with a sense of perceived burdensomeness, sense of belonging is significantly related to suicidal ideation in college students (Van Orden et al., 2008). Although sense of belonging may fluctuate in relation to emotional disturbances or current life stressors, individuals who report a history of psychiatric treatment or suicidality but are no longer depressed demonstrate lower levels of belonging than individuals with no history of psychiatric treatment or suicidality, suggesting that belonging is more than a symptom of depression and may be a relatively stable trait (Conner et al., 2007).

The experience of the participants showed sense of belonging has demonstrated significant relationships with suicidal thoughts, highlighting its potential utility in refining assessment of suicide risk. Sense of belonging is conceptualized as an individual’s experience of feeling valued, needed, and accepted by people in his or her social environment. Need for love and belonging provides an important target for assessment and intervention in the treatment and prevention of suicide. Cognitive, behavioural, and interpersonal interventions may help to improve an individual’s sense of belonging and decrease symptoms of depression and hopelessness.

Over all, this phenomenological research helps uncover life processes “for qualitatively identifying intervention strategies and evaluating outcomes”. According to Van Manen (1990) the findings of phenomenological research can encourage health practitioners to be more thoughtful in regards to their responses and with “knowing how to act” based on thoughtfulness.

## Conclusion

The findings suggest that the 16 attempt survivors interviewed. In their experiences, they have talked about suffering and problems that has different dimensions. Which includes mental pain, family, social and economic factors, and the need for understanding and loving.

Findings suggest that suicidality cannot be understood from only one perspective, whether this is the dominant narrative or not: clinicians and policy makers need to remain open-minded about how attempt survivors might view their experiences.

This important information can help us develop strategies to prevent suicide among the adult population and to provide support for suicide attempt survivors.

Mental health professionals and counsellors can use the results of this study to effectively support adult suicide survivors. A rich comprehension of survivor experiences can assist and empower mental health professionals to provide, effective psychoeducation and interventions and informed support to suicide survivors.

## Limitations

This study has some limitations. While interpretive hermeneutic phenomenological requires smaller sample sizes to allow for the collection of in-depth data about lived experience and the complex contextual factors that shape it, the generalizability may be limited. Nonetheless, our findings can be generalized only to adult Iranian, and attitudes may differ in other countries or even in other regions of Iran. However, our methodological precautions assure the trustworthiness of our findings. Because the socio-cultural environment has a strong influence on suicidal behaviours, further research needs to be conducted to compare and integrate perspectives from several countries.

## Implications

Results of the study provided important implications related to suicide prevention efforts on adult.

Implications for research and practice.
